# BMI Change After Parent’s Death in Adulthood: Examining Age and Race Differences With Time-Varying Effects Modeling

**DOI:** 10.1093/geroni/igab046.3146

**Published:** 2021-12-17

**Authors:** Hye Won Chai, Hyungmin Cha, Debra Umberson

**Affiliations:** The University of Texas at Austin, Austin, Texas, United States

## Abstract

Parental bereavement in adulthood is a stressful event that can have adverse health consequences for middle and older adults, including weight gain. Considering that the impact of bereavement is found to vary depending on the timing of death as well as across race/ethnicity, changes in weight after a parent’s passing may also be contingent on the timing of parent’s death and the bereaved individual’s race/ethnicity. Using Time-Varying Effects Modeling (TVEM), this study examined whether changes in BMI following a parent’s death differed across respondent’s age when their parent passed away. We also tested whether these age differences varied by race. Data came the Health and Retirement Study (HRS) Waves 1 – 13 and we selected respondents who experienced passing of either parent while participating in HRS. Analyses were run separately for mother’s death (n = 6,191) and father’s death (n = 3,301). Results showed significant racial/ethnic differences in BMI change following a mother’s death, particularly during late midlife to early late life. Specifically, non-Hispanic White and Black adults showed a greater increase in BMI compared to Hispanic adults. These race differences were consistent for father’s death as well, but to a lesser extent compared to mother’s death. Results suggest that White and Black adults who lost their parents between late midlife and early late life gained more weight compared to their Hispanic counterparts. This may be attributed to the racial/ethnic differences in health behaviors in response to parent’s death.

